# Myocardial fibrosis in athletes—Current perspective

**DOI:** 10.1002/clc.23360

**Published:** 2020-03-19

**Authors:** Łukasz A. Małek, Chiara Bucciarelli‐Ducci

**Affiliations:** ^1^ Department of Epidemiology, Cardiovascular Disease Prevention and Health Promotion National Institute of Cardiology Warsaw Poland; ^2^ Msc in Sports Cardiology St. George's University of London London UK; ^3^ Bristol Heart Institute, Bristol National Institute of Health Research (NIHR) Biomedical Research Centre University Hospitals Bristol NHS Trust and University of Bristol Bristol UK

**Keywords:** cardiac magnetic resonance, exercise, late gadolinium enhancement, physical activity, T1‐mapping, training

## Abstract

Several previous studies suggested that prolonged and extensive physical activity might lead to increased prevalence of myocardial fibrosis in athletes. The review summarizes these studies focusing on common patterns of myocardial fibrosis observed in athletes, their potential causes and significance. It also presents recent research on parametric imaging shedding new light on diffuse myocardial fibrosis in athletes. Finally, it reviews how these traditional and novel cardiac magnetic resonance (CMR) techniques can be incorporated in the diagnostic work up to differentiate athlete's heart from cardiomyopathies.

## 1 INTRODUCTION

Studies have shown that prolonged endurance exercise leads to marked elevation of myocardial necrosis markers, brain natriuretic peptides and an increase of inflammatory markers.^1^, ^2^, ^3^ The raise of biochemical markers is directly proportional to the duration and intensity of exercise and inversely proportional to the amount of prior training.^1^ Some imaging heart studies, but not all,^4^ have shown that biochemical changes were accompanied by a decrease of cardiac performance, particularly in relation to the right ventricle^5^, ^6^ and by signs of myocardial oedema.^6^ These alterations were shown to be only transient and do not persist in a longer perspective.^5^, ^6^ Importantly, none of those studies have shown any myocardial fibrosis related to the studied endurance events.^4^, ^5^, ^6^ It was suggested that increase of troponin levels is due to release of cytosolic fraction of this marker without compromise to cardiomyocates or that it is related to muscular fatigue or even transient renal impairment and decreased troponin clearance.^1^ However, other studies demonstrated that some lifelong endurance athletes might be at increased risk of ventricular arrhythmias originating from the right ventricle.^7^, ^8^ These observations were followed by a group of studies suggesting increased prevalence of fibrosis in veteran athletes in comparison to sedentary controls of the same age, leading to the hypothesis that prolonged endurance training, especially without adequate recovery may predispose to myocardial fibrosis.^8^, ^9^ Apart from the risk of arrhythmias, the consequences of myocardial fibrosis may include increased myocardial stiffness and local cardiac dysfunction.^10^

The aim of this review is to summarize current understanding of the possible causes and significance of observed myocardial fibrosis in athletes with new insight from recent studies on diffuse interstitial myocardial fibrosis.

## 2 CMR IN THE ASSESSMENT OF FIBROSIS IN ATHLETES

### 2.1 Focal fibrosis

The detection of myocardial fibrosis with CMR is performed after the administration of a gadolinium‐based contrast agent into an antecubital vein. The gadolinium‐chelates contrast agents used in CMR are mainly extracellular contrast agents, which have an initial relative short vascular phase, followed by an extracellular phase in which the contrast agent diffuses in the interstitium. There is a typical “washin” and “washout” contrast dynamics in different tissues, including the myocardium.^11^ In the normal heart, the interstitial space undergoes normal “washout” of the contrast agent with no contrast accumulation. In the presence of myocardial injury or disease, the extracellular space increases leading to delayed “washout” and contrast accumulation. Higher concentration of contrast agent decreases T1 relaxation time of the studied tissue, thus changing its signal intensity, which appears “bright” (hyperintense) as opposed to the normal myocardium (hypointense). The technique used to image the heart post contrast is called late gadolinium enhancement (LGE) and the images are acquired 10 to 15 minutes postcontrast injection. The two main LGE patterns are (a) “ischemic” type characterized by subendocardial to transmural LGE corresponding to territories of coronary artery supply and (b) “nonischemic” type characterized by mid‐wall or subepicardial location, either focal or patchy, as observed in a range of cardiomyopathies, myocarditis or nonischemic myocardial injury from other causes. While LGE can detect focal myocardial fibrosis, the technique cannot detect interstitial fibrosis.

### 2.2 Diffuse fibrosis

Recent developments in CMR were focused on introduction to clinical practice of sequences called CMR relaxometry (native T1‐mapping and extracellular volume [ECV] of distribution), which is able to detect diffuse fibrosis.^12^ This technique is based on mapping of T1‐relaxation time of myocardium (measured in milliseconds), which varies in relation to the composition of myocardium and rises with the increase in the amount of fibrotic tissue. Registration of T1‐relaxation time maps before and after contrast administration and knowledge of blood viscosity affecting the T1 time (measured by patient's hematocrit) permit calculation of ECV of the myocardium. As native (without contrast) myocardial T1‐mapping is believed to vary substantially between various scanners, different contrast agents and T1‐mapping sequences, the ECV is believed to adjust for most of these differences and to most reliably depict diffuse fibrosis.^12^, ^13^, ^14^ So far, most of the studies on fibrosis in athletes with means of CMR were based only on LGE detection^9^, ^15^ but more recently there are studies incorporating T1‐mapping and ECV calculation.^16^, ^17^, ^18^, ^19^, ^20^, ^21^, ^22^, ^23^ Findings from these studies will be summarized below.

## 3 COMMON PATTERNS OF MYOCARDIAL FIBROSIS IN ATHLETES

Most of the studies on myocardial fibrosis in athletes were based on middle age or veteran endurance athletes, both amateur and professional.^9^, ^15^ These athletes are typically between 30 and 60 years of age, predominantly male and have been engaging for at least 5 to 10 years in mainly endurance (running, biking, or combined) exercises. The presented results should be therefore interpreted in this context.

Detailed analysis of studies on LGE detection in athletes has been presented in earlier reviews.^9^, ^15^ Therefore, here, only a concise summary of the observations will be provided. In general, in asymptomatic endurance athletes with normal ECG three LGE patterns can be distinguished: two nonischemic and one ischemic. The nonischemic patterns include: (a) mid‐myocardial LGE in the insertion points (points in the interventricular septum where right and left ventricle muscles connect called otherwise “hinge points” or junction points) and (b) subepicardial or mid‐myocardial LGE in the inferolateral segments or less commonly in the interventricular septum or elsewhere.

### 3.1 Insertion point fibrosis

**Figure 1:**
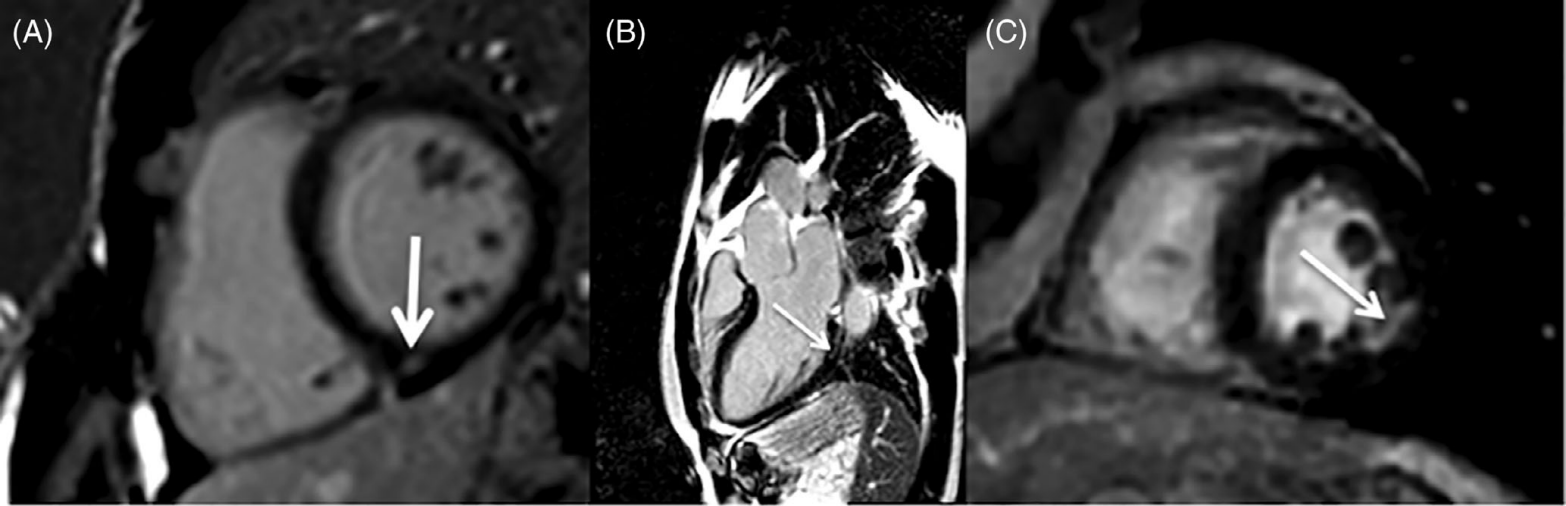
Most common patterns of late gadolinium enhancement (LGE) observed in athletes. A,Short axis view, mid‐myocardial (nonischemic) LGE in the inferior insertion point in an asymptomatic 41‐year‐old ultramarathon runner without prior medical history (own data), B, three‐chamber view, mid‐myocardial (nonischemic) LGE in the basal inferolateral segment in an asymptomatic 41‐year‐old ultramarathon runner without prior medical history (own data), C, short axis view, subendocardial (ischemic) LGE in the mid inferolateral segment in an asymptomatic 52‐year‐old recreational runner without prior medical history (own data)

Insertion point fibrosis is most often limited to the inferior insertion point (Figure 1A). It is the most commonly observed pattern in athletes irrespective of age.^9^, ^15^ Its prevalence may reach up to 20% to 30% and has been correlated with a cumulative training load and training intensity.^24^ These correlations may reflect the time of pressure and/or volume overload present in the right ventricle during intensive exercise, which causes tension on the insertion points and may lead to microinjuries visible later as spots of LGE in that location.^22^ In line with this, similar pattern of fibrosis (however more likely to involve both upper and lower insertion points) has been also observed in patients with pulmonary hypertension or pulmonary regurgitation after repair of tetralogy of Fallot.^25^, ^26^, ^27^ Importantly, in both of those instances, this type of LGE was shown to be benign. Another hypothesis suggests that insertion point fibrosis may be related to myocardial hypertrophy visible in some athletes. Accordingly, a similar pattern of fibrosis is also visible in patients with hypertrophic cardiomyopathy, but a large study found that contrary to more pronounced LGE pattern, these small spots of insertion point fibrosis also do not affect prognosis.^28^ Insertion point fibrosis has been also observed in around 10% of otherwise healthy elderly individuals and may form one of the elements of an aging heart.^22^, ^29^ A recent study has found that in subjects without additional evidence of cardiac damage insertion point fibrosis is to be considered an incidental finding.^30^

### 3.2 Inferolateral or septal nonischemic fibrosis

Inferolateral and septal nonischemic fibrosis is less often observed in athletes than insertion point fibrosis (Figure 1B). Inferolateral mid‐myocardial fibrosis has been previously noticed in some rare storage diseases as Fabry disease,^31^ but is most characteristic of an acute or healed myocarditis.^11^ It is therefore plausible that small, linear subepicardial, or mid‐myocardial areas of LGE in the inferolateral segments or in the interventricular septum in asymptomatic endurance athletes reflect previous, usually silent, myocarditis. Intensive, prolonged exercise has been shown to affect immune resistance in the short period after intensive exercise, which if combined with seasonal infections may predispose athletes to myocarditis.^32^, ^33^ Although this has been so far shown only in animal models, there are case reports supporting this hypothesis.^34^, ^35^ Myocarditis usually resolves without complications and small remnant scars are considered benign and do not require further testing. Such small, silent, mid‐wall areas of fibrosis have also been found in almost 4% of general population.^36^ Only if they are larger and form striae of LGE, particularly in the anteroseptal location, they may increase the risk of severe arrhythmias and affect prognosis as demonstrated in several studies^37^, ^38^, ^39^, ^40^. However, athletes with large areas of postmyocarditis LGE are more likely to be symptomatic and report palpitations or reduced physical performance or present ECG and echocardiographic changes.^37^, ^38^, ^39^

In fact, recent study in a large community‐based sample of older adults from Reykjavik has demonstrated that such minor nonischemic fibrosis patterns, as described in asymptomatic athletes, do not influence prognosis when adjusted for traditional risk factors.^41^

### 3.3 Ischemic fibrosis

Ischemic fibrosis has been reported predominantly in athletes above 50 years of age (veteran athletes) and may reflect the lifelong influence of common cardiovascular risk factors present in these individuals (Figure 1C). Exercise has many beneficial effects on the heart but does not eliminate the presence of all risk factors of atherosclerosis. In fact, the prevalence of common cardiovascular risk factors in Olympic athletes was surprisingly high including 0.3% of hyperglycemia, 3.8% of hypertension, 8% of smoking habit, 18% of positive family history, 25% of increased waist circumference, and 32% for dyslipidemia.^42^ In that study, endurance athletes had generally low cardiovascular risk profile, but one to two risk factors were still present in 50% of them and 2% of them had three to four risk factors. A recent study demonstrated that veteran athletes have more coronary atherosclerotic plaques than age and sex‐matched sedentary controls.^43^ These plaques may arise from increased shear stress during periods of intensive exercise with elevated coronary pressures or might be caused by transient periods of inflammation. However, the morphology of plaques seems more stable (calcified) with lower prevalence of the soft, vulnerable plaques, which are prone to rupture.^43^ Nevertheless, as coronary plaques are present it seems logical that at least some of them may occasionally erode causing small, silent myocardial infarctions (Figure 1C). In fact, silent myocardial infarctions were found not only in veteran athletes, but also in normal population. The results of the MESA study have shown subendocardial (ischemic) scars on CMR in 7.9% of people aged 68 *±* 9.^36^ In most of them (78%), these scars were not visible on electrocardiogram or by clinical adjudication. Exploring the prevalence and size of silent ischemic scars in veteran athletes and age and sex‐matched sedentary controls could clarify whether the scar burden is smaller in athletes. There is another potential confounding factor, which should be added to the equation—heterogeneity of the veteran athlete group. Some of those athletes are lifelong sportsmen, while others engaged in regular exercise in their mid‐life. It is therefore possible that some events like the diagnosis of risk factors or even an adverse cardiovascular event at young age in a close family member may have influenced their decisions. This would form a considerable inclusion bias, which needs to be considered in future studies.

**Figure 2:**
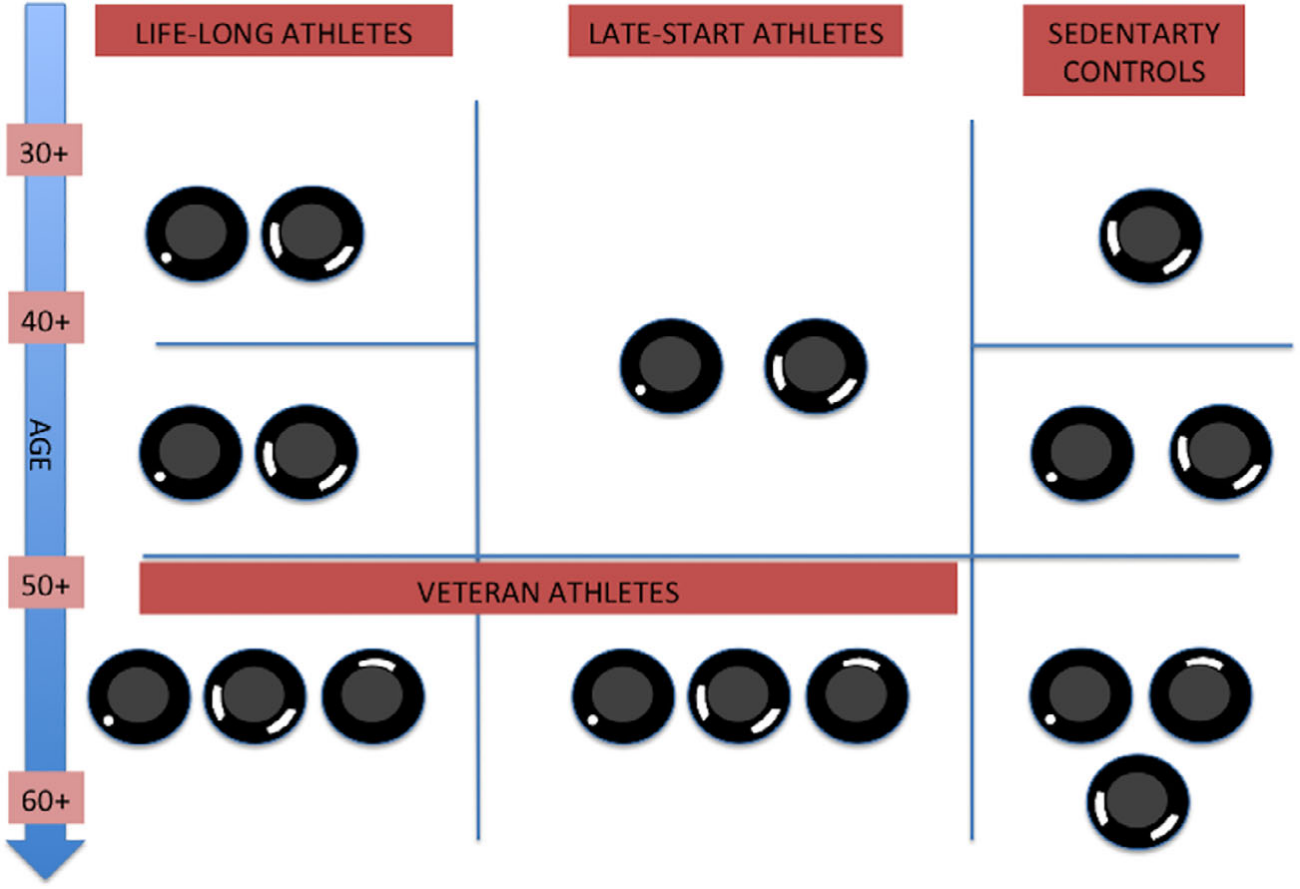
Most common patterns of late gadolinium enhancement (LGE) observed in athletes with different training history and in sedentary controls in relation to age. All drawings of the left ventricle are in short axis. Three groups are presented: on the left—lifelong athletes who start early in life mainly during adolescence (<20 years of age) and eventually become active or sedentary veteran athletes; in the middle—athletes who start later in life (>20‐30 years of age) and continue to become veteran athletes; on the right—sedentary controls. Patterns of fibrosis are described in text and presented in Figure 1. Studies demonstrate that most common patterns of fibrosis in athletes are insertion point fibrosis and myocarditis‐type fibrosis.^16^, ^18^, ^19^, ^21^, ^22^, ^24^ However, both of those patterns were also found in sedentary individuals.^22^, ^29^, ^30^, ^36^ Insertion point fibrosis seems both age and training related and therefore may occur earlier in athletes.^5^, ^22^, ^24^ Ischemic fibrosis was occasionally found with similar frequency in veteran athletes^9^ and sedentary individuals^36^

In summary, the most common patterns of LGE observed in athletes with different training history and in sedentary controls in relation to age are summarized in Figure 2.

## 4 NEW DATA FROM PARAMETRIC IMAGING STUDIES

Only a few recent studies on diffuse fibrosis in athletes can be found.^16^, ^17^, ^18^, ^19^, ^20^, ^21^, ^22^, ^23^ They are summarized in Table 1. In general, there is a large discrepancy between those studies in terms of scanners and sequences used to analyze T1‐mapping, as well as in the use of contrast agent and therefore concomitant LGE reporting (some were based only on native T1‐mapping).^17^ The native T1 time in athletes varies from 943 to 1268 ms and ECV from 20% to 26%. Therefore, each result should be interpreted in relation to internal control group of healthy individuals and dedicated reference values.^12^, ^13^, ^14^ Most of the studies demonstrate that endurance exercise does not lead to an increase of ECV or is related to only slight increase, with absolute values remaining within the reference range for the normal population.^18^, ^19^, ^20^, ^21^, ^22^ Two studies reported a decrease of ECV in athletes, which is explained by increase of cardiomyocyte mass rather than ECV in relation to training.^16^, ^23^ In one of those studies, ECV inversely correlated with increasing left ventricular mass.^16^ Interestingly, one study reported increased ECV values in the remote myocardium of athletes with concomitant LGE, suggesting that fibrosis might not be limited to the macroscopically visible areas of nonischemic LGE in these individuals.^19^

**Table 1:** CMR studies on diffuse fibrosis in athletes with means of T1‐mapping technique and ECV calculation

Study	Study size	Training volume/intensity	Age and sex	LGE vs controls	LGE pattern	T1 vs controls (ms)	ECV vs controls (%)	T1 and ECV in athletes and comments
Malek et al^22^	30 middle age athletes vs 10 controls	Active, median 6 y of ultramarathon running	40.9 ± 6.6, 100% male	27% vs 10%	Nonischemic (insertion point—one in control group, lateral wall)	1200 ± 59 vs1214 ± 32, *P* = .33	26.1 ± 2.9% vs 25.0 ± 2.5%, *P* = .29	Similar T1 and ECV
Pujadas et al^21^	34 veteran athletes vs 11 controls	>10 y of training, still in regular training	48.2 ± 7.5, 100% male	9% vs 0%	Nonischemic(insertion point, lateral wall)	943.6 ± 53 vs 984 ± 37, *P* = .006	25.0 ± 2.0% vs 22.0 ± 2.0%, *P* < .001	Lower T1 and higher ECV, but not after correction for hematocrit
Banks et al^20^	40 athletes vs 8 controls	10 y of competitions, currently 5.2 ± 2.6 h/wk	54 ± 5, 100% male	—	—	1172 ± 29 vs 1187 ± 19, ns	20.7 ± 3.7% vs 17 ± 1.9%, *P* < .05	Similar T1 and higher ECV, values within normal range in both groups
Gormeli et al^17^	46 athletes vs 41 controls	Two groups > and <5 y of sport activity, around 8.6‐9.5 ± 2.5 h/wk	24.5 ± 3.05, 62.2% male	—	—	1268 ± 48 vs 1180 ± 27, *P* < .001	—	Higher T1, no ECV calculated
Treibel et al^23^	50 athletes vs 30 healthy volunteers	>10 endurance events in lifetime	42 ± 14 y, 80% male	Those with infarct pattern not included, other types not reported		—	26.2 ± 2.7% in young athletes vs 28.0 ± 2.9%	Lower ECV
Tahir et al^19^	83 athletes vs 36 controls	>3 y of competitions, >10 h/wk	43 ± 10 y, 65% male	17% male, 0% female vs 0% ns	Nonischemic (inferolateral, insertion points)	Male 990 ± 28 vs 1014 ± 28,*P* < .01 Female 1015 ± 25 vs 1059 ± 22, *P* < .0001	Male 24.8 ± 2.2% vs 24.0 ± 3.0, ns Female 27.8 ± 1.9% vs 28.9 ± 3.3, ns	Lower T1 and similar ECV Athletes with LGE had higher remote myocardium ECV
McDiarmid et al^16^	30 athletes vs 15 controls	Athletes committing on regional, national, or international level	31.7 ± 7.7 y, 100% male	3% vs 0%	Nonischemic (postmyocarditis pattern)	1178 ± 32 vs 1202 ± 33, *P* = .02	22.5 ± 2.6% vs 24.5 ± 2.2%, *P* = .02	Lower T1 and ECV
Mordi et al^18^	21 athletes with depressed LVEF vs 21 controls	>6/h per wk of intensive aerobic exercise at an amateur level	45.9 ± 10.7 y, 100% male	9.5% vs 0%	Nonischemic (insertion points)	957 ± 32 vs 952 ± 31, ns	26.3 ± 3.6% vs 26.2 ± 2.9, ns	Similar T1 and ECV

Abbreviations: ECV, extracellular volume; LGE, late gadolinium enhancement; LVEF, left ventricular ejection fraction.

## 5 MYOCARDIAL FIBROSIS IN ATHLETES; DISTINGUISHING NORMAL FROM ABNORMAL PATTERNS

All of the above considerations were based on observations made in asymptomatic athletes. However, CMR is currently a well‐established second‐line imaging test (after echocardiography) in athletes with specified cardiomyopathy. Several features differentiating athlete's heart and various cardiomyopathies have been proposed and are summarized in Table 2 along with CMR characteristics of the athlete's heart.^44^, ^45^ In all conditions LGE detection and its pattern may be of paramount relevance to the diagnosis and management. Recently, also parametric imaging including T1‐mapping and ECV calculation have been used to detect diffuse fibrosis in at least some cardiomyopathies such as hypertrophic cardiomyopathy (HCM) and dilated cardiomyopathy.^12^ Presence of LGE in locations not observed in athletes or larger areas of LGE together with increase in T1‐mapping time and ECV are some of the features, which may help to differentiate cardiomyopathy from the athlete's heart.

**Table 2:** Typical CMR features, including LGE and T1‐mapping, used in differentiation of athlete's heart from cardiomyopathies (modified from references^44^, ^45^)

	CMR characteristics
Athlete's heart	Fibrosis: Possible insertion point LGE, normal or reduced T1 time and ECV Other features: Symmetric enlargement of all heart chambers (balanced chamber mild dilatation), high bilateral stroke volumes, concentrically increased myocardial thickness usually up to 13 mm
HCM	Fibrosis: Mid‐myocardial LGE in the hypertrophied segments, increased T1 time and ECV Other features: Asymmetric hypertrophy > 13 mm, small LVEDd< 54 mm, more prominent left atrial enlargement, multiple myocardial clefts/crypts
DCM	Fibrosis: Nonischemic patterns of LGE in the LV, increased T1 time and ECV Other features: LVEDd> 60 mm, increased LV volume asymmetrically to other chambers, reduced LVEF not significantly increasing or decreasing during exercise
ARVC	Fibrosis: Nonischemic patterns of LGE in the LV Other features: Regional RV wall akinesia/dyskinesia or dyssynchrony plus RVEDVi meeting major TFC for ARVC or RVEF < 40%, disproportionally larger RV than LV
LVNC	Fibrosis: Nonischemic patterns of LGE in the LV Other features: Noncompacted to compacted layer ratio >2.3 (measured in long‐axis view avoiding the apex), reduced thickness of the compacted layer, involvement of several LV segments, LVEF < 50%

Abbreviations: ARVC, arrhythmogenic right ventricular hypertrophy; DCM, dilated cardiomyopathy; ECV, extracellular volume; HCM, hypertrophic cardiomyopathy; IVS, interventricular septum; LGE, late gadolinium enhancement; LV, left ventricle; LVEDd, left ventricular end‐diastolic diameter; LVEF, left ventricular ejection fraction; LVNC, left ventricular noncompaction; RV, right ventricle; RVEDVi, right ventricular end‐diastolic volume index; RVEF, right ventricular ejection fraction.

If fibrosis is detected we propose the following management strategy. Only the detection of small insertion point fibrosis does not seem to require further evaluation. Presence of fibrosis extending beyond the insertion points in the interventricular septum or fibrosis elsewhere in the myocardium regardless of its pattern should prompt further evaluation, especially in the younger athlete age group. The index of suspicion, regarding potential cardiac disease, should increase if abnormal ECG or the presence of arrhythmias accompanies the detection of fibrosis.

## 6 GAPS IN RESEARCH

Further studies are needed to comprehensively analyze not only the presence, but also the relation between different patterns and amount of LGE in asymptomatic athletes and arrhythmia burden. Also, there is no data on factors potentially predisposing athletes to fibrosis including certain types of training, number of years or intensity of exercise or sex predominance. Furthermore, it should be acknowledged that the studies presented in the review are all cross sectional in nature. There is still a need for large longitudinal studies with clinical end points to draw final conclusions.

## 7 CONCLUSIONS

Studies on myocardial fibrosis in endurance athletes demonstrate that this form of sport activity does not lead to diffuse fibrosis of the myocardium. However, in some athletes, nonischemic foci of fibrosis may be observed, but are mainly limited to insertion points. Endurance athletes may be also more prone to myocarditis (often silent), which is usually detected by visualization of small nonischemic scars. It is also doubtful that endurance activity increases the risk of ischemic fibrosis beyond that which can be explained by age and the presence of common cardiovascular risk factors. The low risk profile of myocardial fibrosis in otherwise asymptomatic patients is supported by the recent large‐scale observations that a mid‐life cardiorespiratory fitness inversely correlated with long‐term risk of mortality and that intensive and vigorous physical activity does not seem to increase the risk of ventricular arrhythmias.^46^, ^47^

Based on current evidence, increased availability and use of CMR is likely to identify small volume of scar of uncertain significance in a considerable proportion of athletes. As such, CMR should be reserved for individuals with high index of suspicion for cardiac disease, including athletes with clinical symptoms or abnormalities on first‐line investigations, as it may elucidate diagnosis and direct management.

## CONFLICT OF INTEREST

The authors declare no potential conflict of interests.
